# Ligation site of the inferior mesenteric vein in laparoscopic radical resection of rectal cancer: a study protocol for a multicenter randomized–controlled trial

**DOI:** 10.1093/gastro/goag044

**Published:** 2026-05-14

**Authors:** Shidong Hu, Songyan Li, Yang Yan, Lin Hua, Yi Xiao, Tao Li, Xiaohui Du

**Affiliations:** Senior Department of General Surgery, The First Medical Center of Chinese PLA General Hospital, Beijing, P. R. China; Senior Department of General Surgery, The First Medical Center of Chinese PLA General Hospital, Beijing, P. R. China; Senior Department of General Surgery, The First Medical Center of Chinese PLA General Hospital, Beijing, P. R. China; Department of Mathematics, School of Biomedical Engineering, Capital Medical University, Beijing, P. R. China; Division of Colorectal Surgery, Department of General Surgery, Peking Union Medical College Hospital, Chinese Academy of Medical Sciences & Peking Union Medical College, Beijing, P. R. China; Department of Radiology, The First Medical Center of Chinese PLA General Hospital, Beijing, P. R. China; Senior Department of General Surgery, The First Medical Center of Chinese PLA General Hospital, Beijing, P. R. China

**Keywords:** inferior mesenteric vein, rectal cancer, lymph nodes, surgery, prognosis

## Abstract

**Background:**

The optimal level of ligation of the inferior mesenteric vein (IMV) during rectal cancer surgery remains unclear. Lymph nodes are distributed at the root of the IMV, but whether root ligation and lymphadenectomy significantly increase the number of lymph nodes harvested or improve patient prognosis remains unclear. This multicenter randomized–controlled trial was designed to compare long-term survival and short-term outcomes (surgical complications, lymph node yield, postoperative recovery) between high and low IMV ligation during laparoscopic radical rectal cancer resection.

**Methods:**

This multicenter randomized–controlled trial will enroll 1,516 patients undergoing laparoscopic-assisted radical resection for rectal cancer. Participants will be recruited and randomized (1:1) by using a dynamic minimization algorithm to balance covariates, including age, sex, American Society of Anesthesiologists classification, tumor location, body mass index, preoperative clinical stage, and neoadjuvant therapy. Patients will be allocated to either high ligation or low ligation of the IMV. The primary endpoint is 3-year disease-free survival.

**Discussion:**

The IMV ligation level is a critical yet controversial step in radical rectal cancer resection. High-level evidence is lacking regarding whether high IMV ligation improves rectal cancer prognosis, necessitating robust evidence to determine the optimal ligation level.

**Trial registration:**

ChiCTR, ChiCTR2300069149. Registered 8 March 2023, https://www.chictr.org.cn/showproj.html? proj=187562.

## Introduction

Colorectal cancer (CRC) has a high global incidence, ranking as the third-most common malignancy worldwide according to the latest World Health Organization data, with ∼915,900 deaths annually. Surgery remains the cornerstone of CRC treatment [1]. The optimal level of ligation of the inferior mesenteric vein (IMV) during rectal cancer surgery remains unclear. European and American scholars advocate high ligation at the lower pancreatic border, aligning with central vascular ligation principles to minimize tumor dissemination, enhance lymphadenectomy at the vascular root, and facilitate tension-free intestinal anastomosis [2]. Conversely, Japanese guidelines recommend caudal ligation of the IMV to mitigate intestinal congestion [3]. The classic surgical textbook Huang Jiasi Surgery, Eighth Edition recommends that the disconnected IMV should be exposed on the outside of the root of the inferior mesenteric artery [4].

NCCN guidelines recommend the surgical principle of total mesorectal excision (TME) to dissect the lymphatic drainage area of the tumor and do not recommend routine extended lymphadenectomy [5]. ESMO guidelines emphasize high-quality surgical resection (TME) [6]. The Japanese Guidelines for Diagnosis and Treatment of Colorectal Cancer (8th Edition) do not have special restrictions on the selection of rectal cancer surgery; in principle, TME is used as the benchmark and the scope of the lymphadenectomy is determined by the depth of tumor invasion and preoperative lymph node evaluation, and prophylactic extended lymphadenectomy is generally not performed [7]. The Chinese Guidelines for the Diagnosis and Treatment of Colorectal Cancer (2020 Edition) recommend the removal of lymph nodes and adipose tissue in the mesorectum and lateral lymph nodes that are suspected of being positive [8]. Neither domestic nor foreign guidelines have clarified the method of IMV ligation. It is believed that lymph nodes are distributed along arteries, and the ligation and dissection of lymph nodes at the root of the IMV have not become a standard procedure for TME surgery [9]. However, it has been shown that there is a distribution of lymph nodes in the root of the IMV, and it is unclear whether root ligation and lymphadenectomy can significantly increase the number of lymph nodes harvested and whether they can improve the prognosis of patients [10].

Studies on the effects of IMV ligation levels on short-term and long-term efficacy in patients are rare. Previous studies have reported that, compared with high ligation (at the inferior pancreatic border), surgical outcomes (operation time, intraoperative blood loss, postoperative drainage, hospital stay), oncological parameters (pathological stage, lymph node yield, positive lymph nodes), and functional follow-up scores (postoperative physical score, voiding function score, defecation function score, intestinal function score, and radiotherapy and chemotherapy side effect score) were comparable, while the time to first flatus was shorter (49.30 ± 19.88 vs 67.83 ± 23.00 h, *P *< 0.05) [11]. There is no high-quality evidence-based medical evidence to confirm whether the ligation methods at different parts of the IMV affect the long-term prognosis of patients with rectal cancer.

## Objectives

This multicenter randomized–controlled trial aims to compare long-term survival (primary) and short-term outcomes (surgical complications, lymph node yield, postoperative recovery) between high and low IMV ligation during laparoscopic radical resection for rectal cancer to determine the optimal level of IMV ligation.

## Methods and trial design

### Subjects of the study

The inclusion criteria for participants were as follows: (i) age 18–75 years; (ii) diagnosis of malignant tumors confirmed by colonoscopic pathological biopsy and indicated for laparoscopic radical resection of rectal cancer; (iii) preoperative imaging (pelvic/rectal magnetic resonance imaging [MRI] mandatory) confirming clinical stage I (T2N0M0), II (T3–4N0M0), or III (T1–4N1M0 or T1–4N2M0) on the American Joint Committee on Cancers TNM Staging System for Colorectal Cancer (Eighth edition, 2017); (iv) completion of pelvic/rectal MRI for clinical staging; (v) American Society of Anesthesiologists (ASA) Grades I–III; and (vi) willingness to participate and cooperate with the study. The exclusion criteria were as follows: (i) a history of other malignant tumors and abdominal vascular surgery; (ii) patients contraindicated for laparoscopic surgery (such as those with extensive adhesion caused by a previous major abdominal surgery or patients not suitable for pneumoperitoneum); (iii) patients with intestinal obstruction, perforation, or bleeding and other conditions requiring emergency surgery; (iv) pregnant or lactating women; or (v) patients who planned to participate in other clinical studies.

### Number of research cases

This is a superiority trial. The 3-year disease-free survival (DFS) rates of patients with high inferior mesenteric artery (IMA) ligation were 86.1% and 84.5%, and the 3-year DFS rates of the experimental group (IMV high ligation and left colonic artery D3 lymph node preservation group) in this study were estimated to be 85% [12, 13]. The 3-year DFS rates of patients with low IMA ligation and 253 lymphadenectomy were 72.4% and 83.9%, respectively, and the 3-year DFS rate of the control group (IMV low ligation and left colonic artery D3 node dissection group) was estimated to be 78% [12, 13]. Assuming 3-year DFS rates of 85% (high ligation) and 78% (low ligation), a superiority margin (hazard ratio) of 0.90, *α* = 0.05, *β* = 0.8, and a superiority cutoff value of the risk ratio of 0.90, the calculation was performed by using a two-sided *α* (0.05), and the enrollment time was 2 years according to the ratio of 1:1 between the two groups, assuming that the unit enrollment rate was equal when patients were recruited and that the follow-up time was 3 years. PASS 15.0 software was used for calculations and the loss-to-follow-up rate of both groups was set at 20%; thus, 758 cases are required for each group. The total required sample size is 1,516.

### Patient enrollment process

#### Perioperative management

Bowel preparation: The usual bowel preparation for 1–2 days should be followed.

Antibiotics: Broad-spectrum antibiotics for gram-negative and gram-positive bacteria should be given intravenously before surgery. After the operation, the diet should be gradually resumed according to the patient’s condition. An abdominal drainage tube should be placed during the operation and the extraction time should be determined according to the drainage volume.

#### Preoperative evaluation

Electronic colonoscopy and pathological biopsy confirm the diagnosis; lung computed tomography (CT), abdominal non-contrast and contrast-enhanced CT, and pelvic or rectal MRI (with or without positron emission tomography [PET]-MRI) can be used to preliminarily determine the depth of tumor invasion in the intestinal wall, the metastasis of regional lymph nodes, and the clinical stage of the tumor. Blood tumor markers: carcinoma embryonic antigen and CA19–9. If necessary, CT angiography can be performed to evaluate the presence of the arc of Riolan, abdominal MRI, liver contrast ultrasound, whole-body bone scan, PET-MRI, and other examinations to confirm distant metastases.

### Patient benefits

All enrolled patients will be prioritized and can voluntarily withdraw at any time.

### Major surgical steps

Low-ligation group: IMV ligation at the left colonic artery bifurcation with D3 lymphadenectomy.

#### Step I: Laparoscopic exploration

The patient’s head is low, the feet are high, and the conventional five-hole method is used to establish the pneumoperitoneum and comprehensively explore the abdominopelvic cavity. The abdominopelvic cavity is checked for metastases, adhesions, ascites, and enlarged lymph nodes, among others.

#### Step II: Identification of ureters and dissection of submesenteric arteries and veins

The peritoneum at the junction of the sigmoid colon and rectum is incised, with careful ureter identification and protection. The mesentery of the sigmoid colon is mobilized to the place to be resected and concurrently downstream from the upper rectum and the upper rectum from the mesangium. The left colonic artery is free and preserved, and the left colonic artery is ligated and severed after branching out of the inferior mesenteric artery. The lymph nodes at station 253 are dissected (the origin of the inferior mesenteric artery is the cephalad border, the medial border of the IMV is the lateral border, the left semicolonic artery is the foot border, and the right side of the inferior mesenteric artery is the medial border), and ligation and dissection are performed at the intersection of the IMV and the left colonic artery.

#### Step III: Separation of the rectum

Downstream along the presacral space to the coccyx, both sides of the rectum are freed, the lateral ligaments on both sides of the rectum are removed, the anterior wall of the rectum and the rectum to 2 cm below the tumor are freed with exposure of the intestinal wall, and the rectum is cut off and closed with straight cutting closure.

#### Step IV: Digestive tract reconstruction

A mini-laparotomy is performed, creating the incision layer by layer into the abdominal cavity. The sigmoid colon and rectum are removed, and the sigmoid colon is transected by ≥10 cm proximal to the tumor. To ensure that there is no bleeding at either end of the broken ends, the broken ends of the sigmoid colon are placed with a gastrointestinal stapler against the needle seat, and the purse sutures are tightened and ligated. The pneumoperitoneum is reconstructed, the anus is fully dilated, the circular stapler is inserted transanally, the anvil post is advanced through the rectal stump, with the stapler connected against the needle seat, the stapler is tightened and fired to complete the anastomosis, the integrity of the upper and lower incision margins is checked, and the smooth and tension-free status of the anastomosis are confirmed.

#### Step V: Indwelling drainage

The pelvis is copiously irrigated with normal saline, the wound is checked again for no active bleeding, and a closed-suction drain is placed posterior to the anastomosis to be drawn out and fixed. The abdominal incisions are sutured layer by layer.

High-ligation group: IMV ligation at the pancreatic inferior border with root lymphadenectomy.

Following the steps described for the low-ligation group (including station 253 lymphadenectomy), the IMV is further mobilized cephalad to the inferior border of the pancreas and the root lymph nodes of the IMV are dissected (except above the left colonic artery, and the area along the distribution of the 253 lymph nodes is dissected). The main surgical steps are the same as those described above. The surgeon determines whether to perform radical rectal cancer resection and fistula surgery according to the intraoperative situation.

### Random and blinding

#### Randomization method

Randomization will be performed by using dynamic allocation with minimization. Age (<50, ≥50 years), sex (male, female), ASA score (I, II, III), tumor location (distance from the anal margin of the tumor; high: >10 cm, median: 5–10 cm, low: <5 cm), body mass index (<25.0, 25.0–29.9, ≥30.0 kg/m^2^), preoperative clinical stage (I, II, III), and neoadjuvant therapy (not performed, chemotherapy, radiotherapy, chemoradiotherapy) are assigned random numbers. Random number rule: 0001–1516.

#### Minimization of random formulas

Suppose a clinical trial has *N* treatment arms with *M* important prognostic factors, and the number of prognostic factor levels for each is *n*1, *n*2, … *n*r.

Assuming that a new patient is assigned to the *k*-th treatment group, the disequilibrium index of the whole system is *G_k_*, and the *G_k_* value is obtained by summing the *D_ik_* values of all intergroup imbalance indices at the patient level:


$$G_{k}=\sum_{i=1}^{M}W_{i}\times D_{ik}$$

Note: *i* is the *i*-th prognostic factor, *k* is the *k*-th treatment group, *M* is the total number of prognostic factors, and *W_i_* is the weight factor of prognostic factor *i*.

Let *D_ik_* be the amount of variation among the assignments of any given factor level:


$$D_{ik}=\frac{1}{N}\sum_{k=1}^{N}{\left(X_{ik}-\bar{X}\right)}^{2}$$

Note: *X_ik_* represents the number of subjects in the *k*-th treatment group and represents the mean number of subjects in each treatment group. The $\bar{X}$ group with the smallest *G_k_* value is taken as the target group. If the number of groups with the lowest *G_k_* value is greater than one, then one of the groups with the lowest *G_k_* value is selected as the target group with equal probabilities. To increase the uncertainty of the patient’s assignment to the target group, the probability of bias allocation is *P *= 0.8 to reduce selection bias.

If the result is calculated as *G_B_* > *G_A_*, then the probability that the patient will be assigned to Group A is 0.8 and the probability of being assigned to Group B is 0.2.

#### Blinding

Owing to the nature of surgical interventions, the surgeons and patients cannot be blindly assigned to the allocated ligation level. Outcome assessors (radiologists for imaging recurrence evaluation, pathologists for lymph node assessment) will not be informed of the treatment allocation and will complete evaluations by using standardized, de-identified imaging and pathological data only. Data analysts will be blinded to the treatment allocation.

#### Randomization

**Figure 1:**
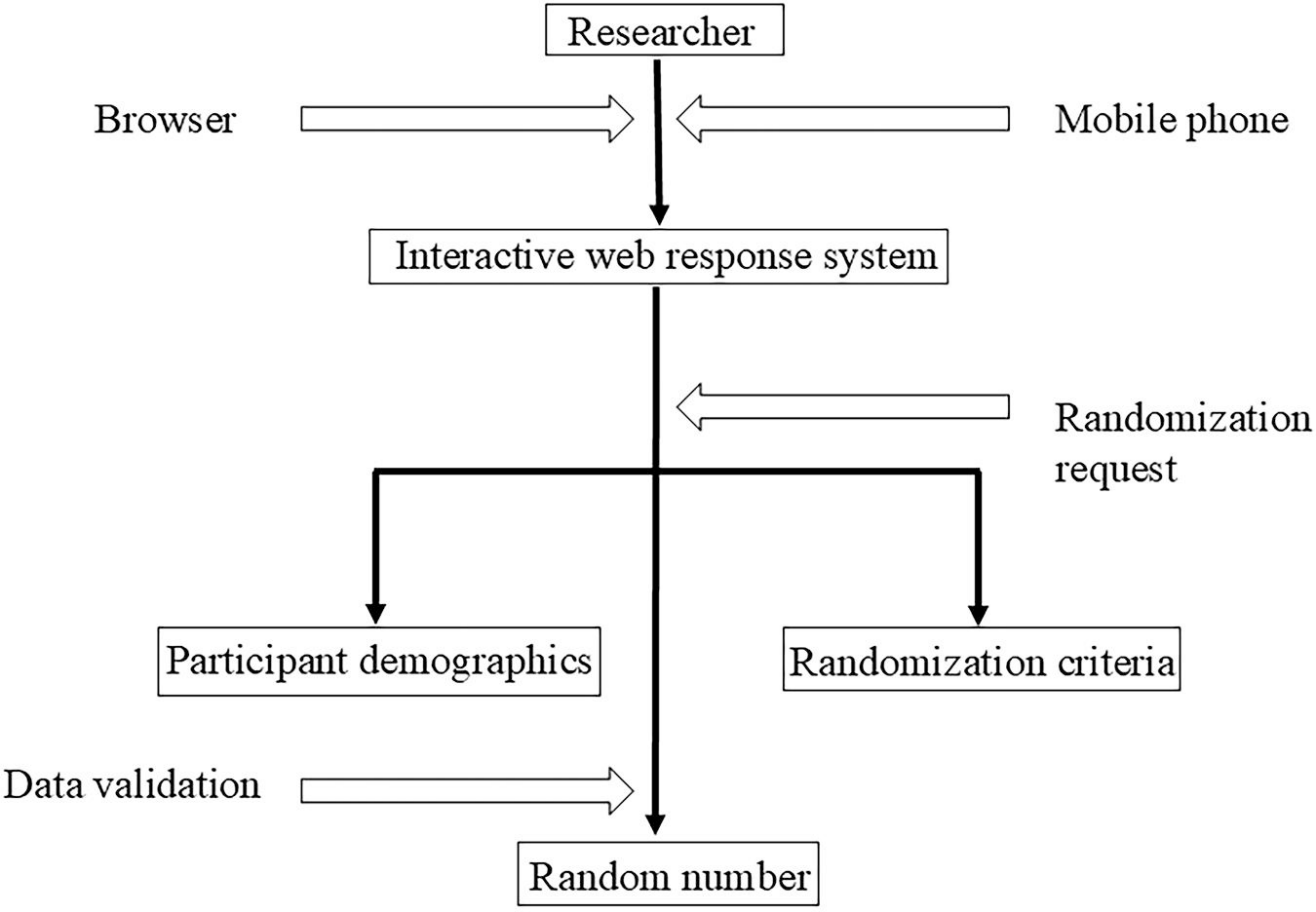
Flowchart of the randomized allocation of study participants. This diagram illustrates the process of randomized participant allocation through an interactive web-based system, comprising two main stages: request and validation.

After the test is completed, the researcher logs in to the data access service (DAS) for the interactive web response system (IWRS) to apply for a random number and prints or downloads the randomization information of the DAS for the IWRS for storage. The stochastic flow chart is presented in Fig. 1.

#### Randomly encoded files

Randomization form: At the end of randomization, the randomization form is exported from the IWRS, submitted to the sponsor, and sealed. Upon study database lock and sponsor approval, a randomization list is generated and provided to the project statistician.

#### Enrollment

In this study, a competitive enrollment method has been adopted and the dynamic minimization method will be used to control the bias of the randomized patients, such as age, sex, ASA, tumor location, body mass index, preoperative clinical stage, and neoadjuvant therapy (the weights of the seven factors are the same; the optimal allocation probability is *P *= 0.8 and the variance will be used to measure the size of the imbalance between the groups), and dynamic minimization enrollment will be achieved via an interactive network response system.

### Study endpoints

Main endpoint indicator: The 3-year DFS rate. Secondary endpoint measures include the following: lymph node yield; operative time; postoperative (30-day) morbidity (anastomotic leakage, incision site infection, postoperative abdominal hemorrhage, gastrointestinal bleeding, intra-abdominal infection, pulmonary infection, urinary system infection, chyle leakage, postoperative intestinal obstruction, cardiovascular event, cerebrovascular event, thrombotic disease, urination disorder, other) and mortality.

Adverse reaction indicators: The occurrence of postoperative complications and treatment measures will be recorded in detail. All adverse events will be recorded and those resulting in hospital admission, prolonged hospital stay, additional surgical or medical treatment, or death will be reported.

#### Metric definitions

DFS: Defined as the time from surgery to tumor recurrence (local or distant) or death from any cause, whichever occurs first, and is assessed monthly.

Recurrence: Inclusive of local recurrence as a recurrence of a tumor in the pelvis or perineum and distant recurrence as a recurrence of a tumor other than in the pelvis.

Local recurrence of the tumor occurring in the surgical area, with or without an increase in tumor markers: a mass found at the anastomosis and identified by pathological biopsy. Distant recurrence of the tumor requires one of the following: signs of local tumor on colonoscopy or barium enema; imaging findings (ultrasound, chest X-ray, CT, MRI, bone scan, PET-CT, etc.) suggestive of recurrence; and the pathological results of needle biopsy at any site consistent with those of the primary tumor. If recurrence cannot be confirmed by imaging, it is considered confirmed by the expert review committee (the person in charge of the First Medical Center of the PLA General Hospital, the participating units including Peking Union Medical College Hospital and Peking University Cancer Hospital).

### Postoperative follow-up

Follow-up will be conducted in accordance with the Chinese Guidelines for the Diagnosis and Treatment of Colorectal Cancer (2020) [8] and will be tailored to patient compliance. The follow-up plan of this study is as follows: (i) History and physical examination and monitoring of carcinoma embryonic antigen and carbohydrate antigen 19-9 every 6 months for 3 years and then annually for a total of 5 years, followed by annually after 5 years. (ii) CT or MRI of the chest/abdomen/pelvis every 6 months for 3 years and then annually for 5 years. (iii) Colonoscopy should be performed within 1 year after surgery. If there is any abnormality, colonoscopy will be reperformed within 1 year; if no polyps are found, reexamination will be performed within 3 years and then once every 5 years. Resection of colorectal adenomas that appear on follow-up examination is recommended. If the colonoscopy does not complete the full-colon examination before surgery, then colonoscopy is recommended to be performed 3–6 months after surgery. (iv) PET-CT is not routinely recommended but may be considered in patients with preexisting or suspected recurrence and distant metastases to further confirm recurrence and metastasis.

More frequent follow-up visits can be performed on the basis of patient compliance; other examinations, such as bone scans and liver MRI, can be improved according to the actual situation of the patient; if it is difficult to complete thoracic/abdominal CT, then chest X-ray and abdominal ultrasound must be completed to confirm the recurrence and metastasis of the liver and lungs.

### Adjuvant and neoadjuvant therapy

In accordance with the Chinese Colorectal Cancer Diagnosis and Treatment Standards 2020 and NCCN (2022 V2), the internal medicine treatment plan of this study was formulated [5, 8].

#### Neoadjuvant therapy

Neoadjuvant chemoradiotherapy is recommended for rectal cancer <12 cm from the anus. Neoadjuvant chemoradiotherapy: Long-course chemoradiotherapy is used and a total dose of 45.0–50.4 Gy is recommended for primary tumors and high-risk areas, with 1.8–2.0 Gy each time, for a total of 25–28 cycles. During radiotherapy, 5-FU or capecitabine monotherapy is given concurrently. The specific situation of neoadjuvant therapy is as follows: (i) Direct surgery is recommended for patients with Tl–2N0M0 or for those with contraindications to radiotherapy and chemotherapy, and neoadjuvant therapy is not recommended. (ii) For patients with resectable rectal cancer with T3 and/or N^+^, preoperative neoadjuvant chemoradiotherapy is recommended in principle; neoadjuvant chemotherapy alone can also be considered after multidisciplinary treatment discussion and then combined with radiotherapy on the basis of efficacy evaluation, with preoperative chemoradiotherapy recommended for patients with stage T4 disease. (iii) For patients who are not suitable for radiotherapy, it is recommended to decide whether to undergo simple neoadjuvant chemotherapy based on multidisciplinary treatment discussions (refer to the Chinese Colorectal Cancer Diagnosis and Treatment Standards 2020).

#### Adjuvant therapy

Adjuvant therapy should be determined on the basis of the patient’s primary site, pathological stage, molecular indices, and postoperative recovery. Adjuvant chemotherapy should be started ∼4 weeks after surgery (appropriately extended for those with poor physical fitness) and the chemotherapy time limit is 3–6 months. During treatment, the dose of the drug should be adjusted and/or the duration of chemotherapy should be shortened according to the patient’s physical condition, drug toxicity, postoperative TN stage, and patient preference. Adjuvant therapy is not recommended for patients with contraindications to chemoradiotherapy:

- I: Adjuvant therapy is not recommended for stage I (T1–2N0M0) rectal cancer.
- II: (i) Adjuvant chemotherapy is recommended for stage II rectal cancer with the following high-risk features: poor histological differentiation (DI or IV) with normal mismatch repair (pMMR) or microsatellite stabilization (MSS), T4, vascular lymphatic invasion, preoperative intestinal obstruction/intestinal perforation, insufficient lymph nodes detected in the specimen (<12), nerve invasion, and positive resection margins. The chemotherapy regimen is CapeOx, FOLFOX, or single-agent therapy such as 5-FU/LV, capecitabine, administered for 3–6 months. There are no risk factors and adjuvant chemotherapy is not recommended. (ii) Postoperative adjuvant chemotherapy is not recommended if the tumor tissue shows mismatch repair deficiency (dMMR) or high-level microsatellite instability (MSI-H).
- III: Adjuvant chemotherapy is recommended for stage III rectal cancer, and CapeOx, FOLFOX, or single-agent capecitabine, 5-FU/LV is recommended for chemotherapy. For low-risk patients (T1–3N1), 3 months of adjuvant chemotherapy with the CapeOx regimen may also be considered.
- IV: For T3–4 or N1–2 rectal cancer located <12 cm from the anal verge, preoperative neoadjuvant chemoradiotherapy is recommended; if there is no neoadjuvant radiotherapy before surgery, whether to undergo adjuvant chemoradiotherapy is decided on the basis of the postoperative pathological situation, among which chemotherapy is recommended to be based on fluorouracil drugs. (i) Patients with stage II–III rectal cancer who have one of the following risk factors after surgery are recommended to undergo concurrent chemoradiotherapy: circumferential resection margin (CRM) of ≤l mm, pT4b, pN2 and poor TME quality/mesorectal defect, pathology II/stage III but unable to evaluate the quality of TME surgery, nerve invasion close to MRF, pathology stage II/III but unable to assess CRM status. (2) Patients with stage II–III rectal cancer after surgery are recommended to receive concurrent chemoradiotherapy if they meet both of the following conditions: CRM of 1–2 mm and circumferential obstructive tumors. (iii) Postoperative concurrent chemoradiotherapy is not recommended for patients with stage II–III rectal cancer who meet all of the following conditions: good TME quality/smooth and intact mesorectum, pT1–3 or pT4a above peritoneal retroflexion, pN0–l, and CRM of >2 mm.

### Subject management

#### Subject exclusion criteria

- After being selected for randomization, the patient requested to withdraw from the study because of various considerations.
- There are no data after entering the queue.
- Intraoperative findings of tumor involvement requiring multivisceral resection, distant metastasis, or inability to complete the planned laparoscopic radical resection.
- Other reasons need to be excluded as determined by the investigator.

#### Criteria and management of subject dropout

##### Shedding criteria

Any randomized participant who discontinues study participation or is lost to follow-up before completing the protocol-specified observation period will be considered a dropout.

##### Dropout treatment

When subjects drop out, the investigator will contact the subjects as much as possible by visiting their homes, making appointments for follow-up, telephone, and letters, among others, asking for reasons and to complete the evaluation items that can be completed. If the experimental case is withdrawn because of adverse reactions, the investigator should take corresponding treatment measures according to the actual situation of the subject. The relevant test data of the expulsion cases are properly maintained, i.e. they are kept as documents, and they are also required for the full analysis set (FAS) statistics. Patients who have dropped out do not need to be replaced.

### Data management

#### Data entry

Data-management personnel: Research assistants and follow-up personnel of each subcenter. Data managers should upload and review research data in a timely manner to ensure data accuracy and their main responsibilities are as follows:

- Recording: synchronous data entry is performed.
- Auditing of data: each item in the database is checked and found to be inconsistent, and the original data are checked and corrected sequentially.
- During the trial, the informed consent of the subjects and the screening of inclusion should be checked regularly.
- Confirm that all case report forms are completed correctly and are consistent with the original data.
- Based on the internet database, real-time monitoring and online quality control are conducted. Once data problems are identified, telephone inquiries are conducted in time to modify the data.
- Withdrawals and loss to follow-up of verified participants are described in the case report form.
- It is confirmed that all adverse events have been documented and that serious adverse events have been reported and documented.

Statistician: Hua Lin. Statisticians are responsible for data collection, collation, and analysis. The details are as follows:

- Locking of the database: after the research is completed, the database will be locked and the locked data are not allowed to change in principle; if changes are required due to special circumstances, the data manager must apply in writing to the person in charge of the study to explain the reason for the application for unlocking, and the person in charge of the study and the statistician shall negotiate to determine whether to agree to the unlocking.
- Data analysis: the database is analysed according to a statistical plan.

### Data retention and summary

#### Data retention

All study data will be retained by the investigators for a minimum of 5 years after trial completion, in accordance with GCP and institutional policies.

#### Summary of the trial

After completion of the trial, all case report forms will be filled out, reviewed, and signed by the research assistant and follow-up staff according to the data-management requirements of this program and submitted to the designated person in charge of each center for preservation together with the “informed consent form.”

### Statistical methods

#### Evaluation and analysis of efficacy

FAS population: This dataset is derived from the minimal and reasonable culling of subjects from all randomized subjects, as close as possible to the population of subjects that conform to the principles of the intention-to-treat (ITT) analysis. ITT: A patient is considered an ineligible study subject and is placed in the ITT population as a result of a change in the assigned surgical regimen or the implementation of an unplanned alternative treatment on the basis of the judgment of the study group or the patient’s special condition. ITT analysis of the primary and secondary endpoints will be performed by using whole-population data.

Eligible for the protocol (PP) population: Subjects in the FAS who meet the following conditions—the baseline value of the main indicators is complete, meets the inclusion criteria and does not meet the exclusion criteria, completes the specified treatment regimen after random assignment according to the clinical trial protocol, and has good compliance. For auxiliary purposes, if the difference between the ITT and PP analysis groups is >10%, then the ITT analysis is used.

#### Safety evaluation and analysis

Safety analysis (SS) population: All subjects who have had at least one safety assessment after randomization should be included. The security analysis should use the security dataset of the analysis population. Safety datasets: Ineffective cases are considered to be the worst-case scenario, such as the evaluation of liver function, which is considered to be the worst, unlike the ITT analysis, which presents the most recent results and may be improved.

It is based mainly on the occurrence of adverse events and serious adverse events, and the population data are analysed with safety analysis.

#### Estimation of missing values for major indicators

When there are missing values in the efficacy and safety evaluation variables, the filling method is appropriately selected according to the missing rate of the main indicators. For missing data in the primary and secondary endpoints, if the missing-data rate is low (<10%), complete-case analysis will be used. If the degree of missing data exceeds 10%, then multiple imputation methods are employed. Sensitivity analyses will be performed to assess the robustness of the results to missing-data assumptions and a sensitivity analysis of the missing mechanism of the main indicators will be conducted.

#### Statistical analysis

Statistical analyses will be performed by using SPSS and systematically categorized according to the study indicators, including baseline characteristics, primary endpoint, and secondary endpoints. An interim analysis upon the enrollment of half of the anticipated sample size will be taken into consideration based on the actual progress of the participant recruitment, clinical implementation status, and data availability. Descriptive analyses of continuous data include the mean, standard deviation, median, interquartile range, and maximum and minimum values. Descriptive analysis will be used for the inclusion rate and composition ratio of the counted data. If the continuous data conform to normality and variance homogeneity, a *t*-test will be used for the comparison of significant differences and the Wilcoxon rank-sum test will be used for nonconformity. The chi-square test will be used for significant differences in count data and the Wilcoxon rank-sum test will be used for significant differences in grade data. Multivariate analysis is performed by using a logistic regression model. Kaplan–Meier analysis is used for DFS, the log-rank test is used for variability, and a Cox regression model is used for regression analysis. *P *< 0.05 will be considered to indicate statistical significance.

### Research ethics

All the participating sites are required to pass the review by the ethics committee of the center and obtain a certificate before they can conduct research. Patients or authorized family members of patients are required to sign informed consent forms prior to patient randomization. An independent Data and Safety Monitoring Board (DSMB) will be established to periodically review the accumulated safety and efficacy data. The trial may be terminated early on the basis of DSMB recommendations for compelling evidence of superiority, futility, or safety concerns.

### Definitions and reporting of adverse events

#### Definitions of adverse events

Adverse events (AEs) were defined as untoward medical events that occur after participants have been enrolled but are not necessarily relevant to this study. The severity of AEs will be determined by the following criteria: mild, did not affect daily activities; moderate, affected daily activities; and severe, lost the ability to perform daily activities. Serious adverse events (SAEs) were defined as those requiring hospitalization for ≥2 months or resulting in death due to surgical complications during the clinical study.

#### SAE reporting

When an SAE occurs, reasonable medical measures must be taken immediately. The physician or research assistant who has confirmed the SAE should report to the research director of the participating institution; the person in charge of the research unit or the acting person in charge of the research unit shall report the SAE to the person in charge of the study within 24 h of the discovery of the SAE, the person in charge of the study shall report the SAE to the ethics committee of the unit, and the ethics committee of the unit shall fully assess the urgency, importance, and impact of the SAE. After evaluation and discussion, the study may be suspended if necessary.

### Quality-control measures

#### Participation in the center screening

It should be ensured that the surgical team of each center has the ability to carry out standardized laparoscopic radical rectal cancer surgery and has performed >50 laparoscopic rectal cancer resections (≥30 procedures annually). We have standardized procedural videos and images available for the participating centers.

#### Enrollment

All the enrolled patients should be screened in strict accordance with the inclusion criteria and the qualified research centers can conduct intraoperative randomization after intraoperative exploration and definitive radical surgical resection to minimize the rejection rate of the enrolled patients.

#### Quality of surgery

Surgeons who participate in the study are required to follow the TME surgical principles (leave sharp scoring under direct vision in the presacral space; keep the pelvic visceral fascia intact; resect the whole mesorectum or make sure that the distal mesorectum of the tumor is not <5 cm and the extent of intestinal resection is ≥2 cm from the distal tumor) to complete radical rectal cancer resection. The following photos will be acquired during the operation to upload to the data system: IMV after free ligation (undissected IMV), after the third station lymphadenectomy, and *in vitro* specimen (including the intestinal tube and mesangium, with ruler or reference placed next to the specimen). In the HL group, 253 lymph nodes and IMV root lymph nodes will be dissected separately and sent for regular pathological examination, and, in the LL group, 253 lymph nodes will be dissected separately and sent for routine pathological examination. Surgeons need to evaluate the quality of the excised *in vitro* specimens. Referring to the concept of the quality grading of rectal cancer gross specimens proposed by Nagtegaal *et al.* [14], the criteria are as follows: (i) high-quality: mesorectal plane resection, intact mesorectum, no defect of >5 mm on the fascia surface of the pelvic visceral layer, no exposed muscularis of the intestinal wall, and a cylindrical appearance; (ii) moderate-quality: mesorectal internal plane resection, intact mesorectal, pelvic visceral fascia surface defect of >5 mm, no muscularis of the intestinal wall, sufficient mesorectal margins, and an approximately conical appearance; (iii) low-quality: myometrial propria plane resection, incomplete mesorectum, pelvic visceral fascia with a defect of >5 mm, and muscularis of the intestinal wall visible and conical in appearance.

The expert review committee (research initiator: the person in charge of the First Medical Center of the Chinese People’s Liberation Army General Hospital; participating members: the person in charge of Peking Union Medical College Hospital and the person in charge of Peking University Cancer Hospital) randomly select the research cases; after discussion, no fewer than two-thirds of the experts determine that the surgical quality meets the research requirements and the cases are qualified to be enrolled. If they do not meet the requirements of the enrolled surgical quality, they are excluded.

#### Postoperative follow-up

Researchers at each research center can establish stable contact with subjects (such as by establishing a WeChat follow-up group) to facilitate follow-up tracking. The research center needs to complete the follow-up and upload the follow-up data in a timely manner according to the follow-up time node, which needs to be completed within 60 days before and after the follow-up time node in the first year of follow-up and within 90 days before and after the follow-up time node in the second and third years of follow-up. For enrolled patients whose follow-up time exceeds the follow-up time window, the expert review committee and statistical experts will discuss and determine the analysis data set to be included in the enrolled cases.

#### Data quality

Each center has one research assistant and one follow-up officer (responsible for data entry and follow-up in the center). The data-entry system can carry out online quality control of the data in real time and the research assistants of each center are responsible for the supervision and quality control of the research data of the center, identification and correction of the problems in the data-entry process in a timely manner, coordination of the research progress, and facilitation of follow-up completion in a timely and effective manner. The follow-up staff of each center will hold a summary meeting every 6 months to analyse the reasons for dropout, discontinuation, and lack of research, and they will correct the deviations. The person in charge of each center will hold a summary meeting once a year. The data of all the enrolled patients will be maintained or copied and backed up and reviewed by the person in charge of each center at the annual summary meeting to ensure that each enrolled patient meets predetermined research procedures.

## Discussion

The complex anatomy of the IMV (receiving venous branches from the sigmoid colon, descending colon, and splenic flexure and typically accompanying the IMA) and its variable lymphatic drainage patterns underlie the ongoing debate regarding the optimal ligation level [15]. Japanese scholars believe that rectal lymphatic drainage is distributed upward along the artery to the root of the IMA [11]. European and American scholars have studied rectal lymphatic drainage by using mercury in cadavers and it is generally believed that rectal lymph drains upward along the artery to the root of the IMA [11]. One prospective study included 100 patients with rectal cancer who underwent anterior sphincter-sparing rectal resection or low anterior rectal resection with high ligation of the IMA and IMV. The results revealed that 63 patients had a lymph node distribution at the root of the IMV and high ligation of the IMV increased the number of lymph nodes dissected [10].

Yuan *et al.* [16] performed an autopsy analysis of 100 adult corpses and divided them into three types according to the traffic branch of the IMV and its sinks. Type I comprises one left colic vein (LCV) and two or three sigmoid veins (SV). According to the confluence of the LCV branches, it is divided into type I1 (the LCV is a single trunk that receives blood from the splenic flexure and descending colon along the way) and type I2 (the upper and lower veins form the LCV, the upper branch holds the blood of the colonic splenic flexure and the upper descending colon, and the lower branch contains the blood of the lower descending colon and the upper sigmoid colon along the way). Type II is composed of two LCVs: the upper branch contains the blood of the splenic flexure of the colon and the upper descending colon, and the lower branch contains the blood of the lower descending colon and the upper sigmoid colon, with one or two SVs flowing directly into the main body. Type III comprises three LCVs, with the upper branch containing the blood of the colonic splenic flexure and the upper descending colon, the middle branch containing the blood of the middle descending colon, and the lower branch containing the blood of the lower descending colon and the upper sigmoid colon, and one or two SVs. There are different positional relationships between the IMV and the left colic artery (LCA): the IMV is located lateral to the LCA; the IMV and LCA cross at the root level of the IMA; the IMV is located on the inside of the LCA and is ≤15 mm away; the IMV is located on the inside of the LCA and is >15 mm away; and the IMV is located on the outside of the LCA and is >50 mm from the root of the IMA [17]. The IMV most commonly drains into the splenic vein, followed by the superior mesenteric vein (SMV). In addition, it may be confluent at the junction of the SMV and splenic vein, the first jejunal vein, and the middle colonic vein [17, 18]. In our study, images of IMV ligation will be collected to facilitate further analysis of the classification/subtypes of the IMV and its potential impact on the findings.

Many European and American professional books recommend high IMV ligation, i.e. ligation and amputation at the lower border of the pancreas, in rectal cancer surgery [19–21]. European and American scholars believe that this method can remove the lymph nodes at the root of the blood vessel and prevent the spread of tumor cells, which is in line with the principle of central vascular ligation. Moreover, this method can increase the freeness of the descending colon and reduce the tension of the anastomosis, which is conducive to intestinal anastomosis. Japanese investigators have reported that there are anatomical lymphatic vessels following the IMV to the pancreas, but the path and intensity of lymphatic drainage have not been determined, and this static lymphatic drainage path does not fully represent the lymphatic drainage path in the clinical sense; thus, ligation and dissection on the tail side of the IMV are recommended to avoid intestinal congestion [22, 23]. Chinese scholars have not yet reached a consensus on the location of IMV ligation and disconnection. The eighth edition of Huangjiasi’s Surgery, a classic surgical textbook in China, suggests that the IMV should be exposed and severed on the outside of the root of the inferior mesenteric artery [4]. Some Chinese scholars have recommended ligation at the root of the IMV, believing that this is in line with the principle of central vascular ligation; others suggest cutting and ligating the IMV at the level of the IMA root [24, 25]. Chinese scholars studied 28 patients who underwent laparoscopic-assisted anterior rectal resection, of whom 12 were ligated with low IMV (ligated and disconnected IMV at the root level of the IMA) and 16 had high IMVs (ligated at the lower edge of the pancreas to dissect the IMV and dissect the lymphoid fat tissue around the IMV). The results revealed that, compared with the IMV at the root-level ligation and dissection of the lower edge of the pancreas, the IMV was similar in terms of surgical indicators (operation time, intraoperative blood loss, postoperative abdominal drainage, postoperative hospital stay), oncological indicators (postoperative pathological stage, total number of lymph nodes, number of positive lymph nodes), and follow-up indicators (postoperative physical score, voiding function score, defecation function score, intestinal function score, and radiotherapy and chemotherapy side effect score), but the postoperative flatus time was shorter (49.30 ± 19.88 vs 67.83 ± 23.00 h, *P *< 0.05) [17]. Previous studies have shown that high ligation and dissection of the IMV can increase the number of lymph nodes obtained and contribute to the lymph node staging of tumors. However, whether this increased lymph node yield translates into a survival advantage remains unclear [26].

The blinding of surgeons and patients is not feasible. However, this approach may introduce bias in postoperative management (e.g. timing of drain removal, discharge criteria) and the assessment of subjective symptoms.

## Conclusions

The optimal level for IMV ligation during radical rectal cancer surgery remains controversial. The IMV anatomy is complex and requires careful intraoperative identification. Current high-level evidence is insufficient to determine whether high ligation improves prognosis compared with low ligation. This multicenter randomized–controlled trial aims to provide robust evidence to guide clinical practice regarding the level of IMV.
